# Supplementary material for: Pharmacotherapy, drug-drug interactions and potentially inappropriate medication in depressive disorders
Source: PLoS One. 2021 Jul 22;16(7):e0255192. doi: 10.1371/journal.pone.0255192 (PMC8297778; doi:10.1371/journal.pone.0255192)
Supplement: Attachment — Submitted filename: Answers to reviewer comments.pdf [file pone.0255192.s001.pdf]

Pharmacotherapy, drug-drug interactions and potentially inappropriate medication in depressive disorders

Answers to reviewer comments, 07. July 2021

Correspondence: Dr. Jan Wolff, Peter L. Reichertz Institute for Medical Informatics of TU Braunschweig and Hannover Medical School, Hannover, Germany. Address: Karl-Wiechert-Allee 3, 30625 Hannover, Mobile: +491702322201, Email: wolff.jan@mh-hannover.de, ORCID: https://orcid.org/0000-0003-2750-0606

Editor, Comment #1

Comment: "*We note that you received funding from a commercial source: Janssen Pharmaceuticals, Inc, Janssen-Cilag and Neuraxpharm, Janssen-Cilag. Please provide an amended Competing Interests Statement that explicitly states this commercial funder, along with any other relevant declarations relating to employment, consultancy, patents, products in development, marketed products, etc.Within this Competing Interests Statement, please confirm that this does not alter your adherence to all PLOS ONE policies on sharing data and materials by including the following statement: "This does not alter our adherence to PLOS ONE policies on sharing data and materials." (as detailed online in our guide for authors http://journals.plos.org/plosone/s/competing-interests). If there are restrictions on sharing of data and/or materials, please state these. Please note that we cannot proceed with consideration of your article until this information has been declared. Please include your amended Competing Interests Statement within your cover letter. We will change the online submission form on your behalf.Please know it is PLOS ONE policy for corresponding authors to declare, on behalf of all authors, all potential competing interests for the purposes of transparency.*"

Our answer: We have adjusted the Competing Interest Statement as follows and included it in our cover letter: *"The commercial funding sources were unrelated to the present study and did not alter our adherence to PLOS ONE policies on sharing data and materials."*

Editor, Comment #2

Comment: "*In your Data Availability statement, you have not specified where the minimal data set underlying the results described in your manuscript can be found. PLOS defines a study's minimal data set as the underlying data used to reach the conclusions drawn in the manuscript and any additional data required to replicate the reported study findings in their entirety. All PLOS journals require that the minimal data set be made fully available. For more information about our data policy, please see http://journals.plos.org/plosone/s/data-availability. Upon re-submitting your revised manuscript, please upload your study's minimal underlying data set as either Supporting Information files or to a stable, public repository and include the relevant URLs, DOIs, or accession numbers within your revised cover letter. For a list of acceptable repositories, please see http://journals.plos.org/plosone/s/data-availability#loc-recommendedrepositories. Any potentially identifying patient information must be fully anonymized. Important: If there are ethical or legal restrictions*

to sharing your data publicly, please explain these restrictions in detail. Please see our guidelines for more information on what we consider unacceptable restrictions to publicly sharing data: http://journals.plos.org/plosone/s/data-availability#loc-unacceptable-data-access-restrictions. Note that it is not acceptable for the authors to be the sole named individuals responsible for ensuring data access."

Our answer: We have adjusted the data availability statement as requested and included it in our cover letter: *"Our study required potentially identifying and sensitive human research participant data, such as psychiatric diagnoses, medication data and many others. Ethical and legal restrictions in Germany and the European Union prohibit sharing these sensitive data. Further information can be obtained from the ethics committee of the State Medical Association of Hesse under the file number FF116 / 2017."*

Editor, Comment #3

Comment: "*Your ethics statement should only appear in the Methods section of your manuscript. If your ethics statement is written in any section besides the Methods, please delete it from any other section."*

Our answer: We have removed the ethics statement from the section of the manuscript where the different statements are listed.

Editor, Comment #4

Comment: *"Please include a copy of Table 1 which you refer to in your text."*

Our answer: We have included a copy of table 1 on page 8 of our new manuscript.

Reviewer #1, Comment #1

Comment: "*The study doesn't analyze the actual consequences of drug interactions. For this reason, the word "potential DDI" should be used instead of DDI, particularly in the results and discussion sections*".

Our answer: We have adjusted the wording in the entire manuscript.

Reviewer #1, Comment #2

Comment: *"Some figures are difficult to understand, and the figure's legends are not clear. I suggest avoiding the insert of data or combination of data that are not relevant to the discussion"*

Our answer: We have reduced the total number of figures by removing Figure 1 and Figure 2. Furthermore, we have added brief statements to each relevant paragraph of the results section that lead to the impetus of including the respective tables and figures in our manuscript. These can be found at the following places of our new manuscript:

Page 10: "*Table 2 shows the determinants of receiving different antidepressant and antipsychotic drug regimes.*

Page 12: "*In clinical practice, knowledge about the relevant drugs and their combinations that account for the respective pDDI and PIM can help the reduce drug safety risks. A relatively large fraction of total cases of pDDI and PIM was accounted for by certain drugs and their combinations. Avoiding these drugs could lead to a substantial reduction of overall cases, as shown in Figure 3.*"

Reviewer #1, Comment #3

Comment: *"in table 1, the variable "Length of stay" is showed with a range and not with a standard deviation"*

Our answer: We have adjusted the line caption of the "length of stay line" in Table 1.

Reviewer #1, Comment #4

Comment: *"in figure 1 is not clear the meaning of the gray bars. Moreover, the differences between groups seem not statistically significant. The figure could be deleted".*

Our answer: We have deleted Figure 1.

Reviewer #1, Comment #5

Comment: *"the drug classes reported in figure 2 are almost the same in the two groups. This figure could also be deleted describing the
most important results in the text"*

Our answer: We have deleted Figure 2 and included the most important results in the manuscript text. The new parts can be found on page 9 of the new manuscript: *"Antidepressants were by far the most frequently used drug group, with 85% of cases with recurrent depressive disorders and 73% depressive episodes receiving at least one drug from this group. The second most frequently used group were antipsychotic drugs, which were used for 61% of cases with recurrent depressive disorders and 52% of depressive episodes."*

Reviewer #1, Comment #6

Comment: *"In part 3A I cannot understand the meaning of the words "at intersection" (number of hospital days?). I suggest using in the figure and the whole text the term Combination AD/AP instead of Augmentation and the term Mono AP instead of Only*

AP. In figure 3C the sum of the percentages is 100% but the group Combination AP is missing."

<u>Our answers:</u>

In part 3A I cannot understand the meaning of the words "at intersection" (number of hospital days?)

We have adjusted the Figure caption (Figure 1 in the new manuscript) and included a description of the word intersection in this context: *"A) Intersection: Number of days with the respective drug or drug combination and the proportion in total patient days. For instance, at 5% of all inpatient days a drug from the class of SSRIs was augmented by a drug from the class of atypical antipsychotics (atyp. AP)."*

"I suggest using in the figure and the whole text the term Combination AD/AP instead of Augmentation and the term Mono AP instead of Only AP"

We would prefer differentiation between the word "combination" for two different antidepressants and the word "augmentation" for the simultaneous use of antidepressants and antipsychotics, since this wording has become the most commonly used definition.

Please see for instance Mouaffak et al.*: "The accepted definition of antidepressant augmentation, validated by international guidelines[1] assumes that* ***augmentation*** *of antidepressants involves adding a second drug, other than an antidepressant, to the treatment regimen when no response or only partial response has been achieved, with the goal of enhancing treatment. Although enhancing the effectiveness of one another, the adding of an antidepressant to an ongoing antidepressant treatment is designated by the neutral generalist terms: association or* ***combination****." (Mouaffak et al., 2014). The same definition is also applied in WFSBP Guidelines for Biological Treatment of Unipolar Depressive Disorders, please see (Bauer et al., 2002).*

Bauer, M., Whybrow, P. C., Angst, J., Versiani, M., Möller, H.-J., & WFSBP Task Force on Treatment Guide. (2002). World Federation of Societies of Biological Psychiatry (WFSBP) Guidelines for Biological Treatment of Unipolar Depressive Disorders, Part 1: Acute and Continuation Treatment of Major Depressive Disorder. *The World Journal of Biological Psychiatry*, *3*(1), 5–43. https://doi.org/10.3109/15622970209150599

Mouaffak, F., Hozer, F., Delomel, O., & Hardy, P. (2014). Adding to Antidepressant Augmentation. *Journal of Clinical Psychopharmacology*, *34*(6), 770. https://doi.org/10.1097/JCP.0000000000000208

Furthermore, we have used the term Only AP to describe both antipsychotic monotherapy and the combination of more than one antipsychotic.

"In figure 3C the sum of the percentages is 100% but the group Combination AP is missing."

We have used the term "Only AP" to describe both antipsychotic monotherapy and the combination of more than one antipsychotic. We have made this more explicit in the caption of the new Figure 1 of our manuscript: "Only AP: Patient received one or more antipsychotic drugs but no antidepressant drugs".

Reviewer #1, Comment #7

Comment: “*'the description of Figure 5 in the results should be changed. The description should list the most involved drugs in each group (e.g. mirtazapine in QT-Combi). The suggestion to avoid certain drugs to substantially reduce the cases should be moved in the discussion with specific reference to the involved drugs.*”

Our answer: We have changed our manuscript and adjusted the description of the former Figure 5, which is now Figure 3. The new parts can be found on page 12 of the new manuscript: *“Figure 3 shows the TOP-20 drugs and drug combinations of each field of pDDI and PIM, respectively. The three most frequently involved single drugs in CYP450-related interactions were Duloxetine, Melperone and Bupropion accounting for 30%, 21% and 17% of all cases affected by CYP450-related interactions. The three most frequently in QT-Combi. involved single drugs were Mirtazapine (42%), Quetiapine (34%) and Pipamperone (28%). The three most frequently in Antichol.-Combi. involved single drugs were Promethazine (49%), Olanzapine (40%) and Amitriptyline (28%). “*

Reviewer #2, Comment #1

Comment: “*The phrases “analyse prescription patterns and determinants of DDI and PMI” is vague. It is recommended that this be rephrased to provide more clarity of precisely what the authors intend to achieve with their analyses”*

Our answer: We have changed this phrase and made it more precise.

The new parts of the manuscript can be found in the new abstract and on page 4 of the new manuscript: *“The aim of this study was to describe the number and type of drugs used to treat depressive disorders in inpatient psychiatry and to analyse the determinants of pDDI and PIM.”*

Reviewer #2, Comment #2

Comment: *“The introduction includes a lot of detail on the specifics of pharmacokinetic and pharmacodynamics of types of drugs without a clear and cohesive argument for how this is relevant to the aim of the study.”*

Our answer: We have adjusted the introduction, reduced these details to one relatively short paragraph and made the importance for the treatment of depressive disorders clearer.

The new parts can be found in our new manuscript on page 3: *“These pDDI are of specific relevance for the inpatient treatment of depressive disorders. Cytochrome P450 (CYP) enzymes are essential for the phase 1 metabolism of drugs and most pharmacokinetic pDDI in the treatment of depressive disorders are the results of inhibition or induction of CYP enzymes [10]. Many drugs for the treatment of depressive disorders have strong anticholinergic effects in connection with their biochemical mechanisms, such as tricyclic antidepressants [11,12]. Drug-induced prolongation of*

the QT interval is associated with an increased risk of a rare but potentially fatal form of cardiac arrhythmia, so-called "torsade de pointes" (TdP), [13]. A prolongation of the QT interval has been shown for several antidepressants, in particular tricyclic antidepressants and the SSRIs (es-) citalopram [14,15].”

Reviewer #2, Comment #3

Comment: *“Potentially inappropriate medications are not clearly defined and a more cohesive argument is needed for why this is important and relevant to the aim of the study”*

Our answer: We have adjusted our manuscript and improved the definition of potentially inappropriate medications and the reasons why these are and important aspect for the aim of this study.

The new parts can be found on page 4 of our new manuscript: “*Pharmacokinetics and -dynamics change in elderly patients due to the progressive decline in the functional reserve of multiple organs and systems with an influence on drug disposition when aging [18]. Medication is considered as PIM if risks outweigh benefits of better alternatives [19–21]. Drug safety requires that these aspects are taken into account in the treatment of depressive disorders in elderly patients.”*

Reviewer #2, Comment #4

Comment: *“More specificity with respect to “prescription patterns” and “determinants” is needed to understand what the aim of this manuscript and how it addresses concerns with DDI and PMI.”*

Our answer: We have made this point more precise.

The new parts of the manuscript can be found on page 4 of the new manuscript: “*The aim of this study was to describe the number and type of drugs used to treat depressive disorders in inpatient psychiatry and to analyse the patient- and treatment-specific determinants of pDDI and PIM.”*

Reviewer #2, Comment #5

Comment: *“This section would benefit from improved organization and greater detail about the study. Included subheadings such as Data source (i.e. the larger study), Study Sample (i.e. the sample used in the present analysis), Measures (i.e., the different types of drug categories and how they are identified, polypharmacy + definition, and patient characteristics and how they are measured/collected). Any measure reported in a table or results section should be described in the Methods/measurement section.”*

Our answer: We have thoroughly adjusted our methods sections. We have included more sub chapters to improve the structure of this section. Furthermore, we have

included all measures and the ways to obtain these data and their purpose for our study.

The parts can be found in the total methods section and on page 6 of the new manuscript in these new lines: *"We obtained patient and treatment data from the patient administration databases of each treatment site. These data were patient gender, age at admission, length of stay, treatment type (i.e. day-clinic versus regular ward), the Clinical Global Impressions at admission [40] and diagnoses according to the International statistical classification of diseases and related health problems. - 10th revision (ICD-10). These data were used to describe the study sample and to adjust for potential confounders in multivariate models."*

Reviewer #2, Comment #6

Comment: *"The authors state that this is part of a larger study and provided a reference for that study. However a brief 1-3 sentence description of the larger study would provide clarity to the context for the present study"*

Our answer: We have described the larger project im more details.

The new parts can be found in our new manuscript on page 5: *"The present study was part of a larger pharmacovigilance project funded by the German Innovation Funds (OSA-PSY - Optimization of inpatient drug therapy for mental illnesses, grant number 01VSF16009). The German Innovation Funds sponsors innovative projects to improve the quality of medical care provided under the statutory health insurance system. The aim of the larger project was to use daily patient-specific medication data and their dissemination among clinical staff to improve drug safety in inpatient psychiatry. The study was approved by the ethics committee of the State Medical Association of Hesse under the file number FF116 / 2017."*

Reviewer #2, Comment #7

Comment: *"It is not clear whether the sample for this study is a subsample from the larger study or uses the same sample but examines a different set of aims than the larger study. More detail about how the present study sample is derived from the larger study is needed"*

Our answer: We have adjusted our manuscript and made this point more explicit.

The new parts can be found in our new manuscript on page 5: *"The present study analysed a sub-sample of the total research project, namely patients with depressive disorders, i.e. a main diagnoses of F32* or F33*, ICD-10. Previous publications from this research project can be found in the reference list [10,12,14,23–26]."*

Reviewer #2, Comment #8

Comment: *"The paragraph "The study investigated . . ." seems to state three aims, which are different from the Aims stated at the end of the introduction. Aims should be stated at the end of the Introduction section and the Methods section should be used to described how these aims were examined."*

Our answer: We have adjusted this part of our manuscript.

The new parts can be found in our new manuscript on page 5: *"The pDDI analysed by our study were defined as 1) pharmacokinetic pDDI via CYP enzyme inducing and inhibiting drugs and the respective victim drugs (CYP450-Interaction), 2) pharmacodynamic pDDI via the administration of more than one anticholinergic drug and (Antichol.-Combi.) 3) pharmacodynamic pDDI via administration of more than one drug that potentially prolongs the QT-interval (QT-Combi.)."*

Reviewer #2, Comment #9

Comment: *"In the logistic regression model, what is the outcome? What is the main predictor of interest? What other covariates are being adjusted for as potential confounders of the relationship you are aiming to examine?"*

Our answer: We have adjusted this part of our manuscript and made these aspects more precise.

The new parts can be found in our new manuscript on page 7: *"We used multivariate logistic regression models to explain the relationship between patient-specific characteristics and type of antidepressant treatment and the outcome of at least one pDDI and at least on PIM during the hospital stay, respectively."*

Reviewer #2, Comment #10

Comment: *"The author present a lot of very detail results and descriptive tables. However, there is not a clear connection between the data being presented in the tables and figures and how the results are relevant to the aims of the study. Without a clear connection between the Aims, analytic methods, and results presented in the Tables and Figures it is difficult to identify the most relevant findings with respect to the aims of the manuscript. It is recommended that the authors reduce the amount of data presented in terms of both the number of tables and figures and the contents of those table to include only data relevant to a narrower, more focused set of aims."*

Our answer: We have adjusted our results sections thoroughly. We have deleted Figures 1 and 2 and reduced the provided data for the part necessary to fulfil the study aims.

Reviewer #2, Comment #11

Comment: *"It is not clear why the authors choose to make a distinction between "recurrent depressive disorders" and "depressive episodes". Are they authors hypothesizing that DDI or PMI would be more prevalent in one of these types of depression? If so, this should be stated in the aims with a justification in the background section."*

Our answer: We have deleted Figures 1 and 2 (Please see our answer to comment #10) and therefore do not stratify the sample into these subgroups anymore.

Reviewer #2, Comment #12

Comment: *"Similarly, in Figure 1, the mild/other, moderate, and severe stratifications are not defined and the connection between the data presented and the aims of the study are not clear."*

Our answer: We have deleted Figure 1. Please also see our answer to comment #10.

Reviewer #2, Comment #13

Comment: *"There are numerous measure in Table 1 that are not described in the Methods section (e.g. Day-clinic, number of comorbidities). The methods section should make clear how/where all data was obtained and how any summary measures were created. For example, what conditions were counted in the "comorbidities" variable. Additionally, the relevance of these measure to the aims should be clarified. Are they being examined as predictors, potential confounders, or outcomes of interest in the multivariate models?"*

Our answer: We have adjusted our manuscript in the methods section and described these measures and how they were obtained.

The new parts can be found in our new manuscript on page 7: *"We obtained patient and treatment data from the patient administration databases of each treatment site. These data were patient gender, age at admission, length of stay, treatment type (i.e. day-clinic versus regular ward), the Clinical Global Impressions at admission [40] and main diagnoses and all psychiatric and somatic comorbidities according to the ICD-10. These data were used to describe the study sample and to adjust for potential confounders in multivariate models."*

Furthermore, we have described in more detail how these data were used in the multivariate models. The new parts can be found in our new manuscript on page 7: *"We used multivariate logistic regression models to explain the relationship between patient-specific characteristics and type of antidepressant treatment and the outcome of at least one pDDI and at least on PIM during the hospital stay, respectively. "*

Reviewer #2, Comment #14

<u>Comment:</u> *“The terms “combination”, “augmenting”, and “switching” are introduced in Figure 3 without being defined in the methods”*

<u>Our answer:</u> We have adjusted our manuscript and introduced these aspects in our methods section.

The new parts can be found in our new manuscript on page 6: *“We differentiated antidepressant drug regimens between a) monotherapy, i.e. receiving one antidepressant drug, b) switch/trial, i.e. receiving more than one antidepressant or antipsychotic drug but not more than three days in combination, c) antidepressant combination, i.e. receiving more than one antidepressant drug in combination more than three days and d) augmentation, i.e. receiving a combination of antidepressant and antipsychotic drugs more than three days [40].“*

Reviewer #2, Comment #15

<u>Comment:</u> “*The discussion include a very thorough review of guidelines and approaches to pharmacotherapy for depression and quantify the prevalence of each treatment approach (e.g., monotherapy, ADs augmented with antipsychotics). However, the clinical value of this information are not clear. The Discussion would benefit from greater organization focused around the key findings.”*

<u>Our answer:</u> We have restructured the discussion subheading clinical implications and differentiated between cinical Implications of different treatment approaches and clinical implications with regard to pDDI and PIM, since we consider both aspects as relevant for clinical practice and the implications of our study findings. However, the discussion should be much clearer structured now and more accessible.

The new parts can be found in our new manuscript starting on page 14.

Reviewer #2, Comment #16

<u>Comment:</u> *“A lot of very detailed information is presented and it is difficult to identify what pieces of information are most important/relevant. It is recommended that the tables and figures be reduced in terms of total number and content to focus on the most important outcomes of interest.”*

<u>Our answer:</u> We have deleted Figure 1 and Figure 2 of our manuscript and we are now focusing on the most relevant aspects of our work.
